# Low-Intensity Pulsed Ultrasound Attenuates Postoperative Neurocognitive Impairment and Salvages Hippocampal Synaptogenesis in Aged Mice

**DOI:** 10.3390/brainsci13040657

**Published:** 2023-04-13

**Authors:** Qian Wang, Taotao Liu, Huixian Chang, Zhengqian Li, Lei Chen, Xinning Mi, Huayi Xing, Xiaoxiao Wang, Jingshu Hong, Kaixi Liu, Yitong Li, Dengyang Han, Yue Li, Ning Yang, Xiaoli Li, Yingwei Li, Xiangyang Guo

**Affiliations:** 1 Department of Anesthesiology, Peking University Third Hospital, Beijing 100191, China; 2 School of Information Science and Engineering, Yanshan University, Qinhuangdao 066104, China; 3 Beijing Center of Quality Control and Improvement on Clinical Anesthesia, Beijing 101300, China; 4 Department of Rehabilitation Medicine, Peking University Third Hospital, Beijing 100191, China; 5 Research Center of Clinical Epidemiology, Peking University Third Hospital, Beijing 100191, China; 6 State Key Laboratory of Cognitive Neuroscience and Learning, Beijing Normal University, Beijing 100875, China

**Keywords:** low-intensity pulsed ultrasound (LIPUS), delayed neurocognitive recovery (dNCR), synaptogenesis, Piezo1, Calpain1

## Abstract

Postoperative neurocognitive impairment is an urgent problem with global aging accelerating. The prevention and treatment of postoperative neurocognitive impairment have been widely investigated but lack effective strategies. Low-intensity pulsed ultrasound (LIPUS), a non-invasive tool, has shown an effect on neuroprotection, but whether it could attenuate the postoperative neurocognitive impairment and the underlying mechanisms remains unknown. An experimental setup for LIPUS stimulation of the hippocampus was well established. A laparotomy model in aged mice was applied, and a Morris water maze was used to assess cognitive function. RT-qPCR and western blotting were used to detect levels of Piezo1, synapse-associated proteins in the hippocampus, respectively. Immunofluorescent staining was also used to determine the neural activation and Piezo1 expression. The results showed that LIPUS increased synapse-related proteins of the hippocampus and attenuated cognitive impairment in aged mice. Meanwhile, LIPUS suppressed the overexpression of Piezo1 in the hippocampus. We further found that LIPUS promoted Calpain1 activity and increased extracellular regulated protein kinases (Erk) phosphorylation. Our results suggested that LIPUS could improve cognitive impairment and increase hippocampal synaptogenesis through the Piezo1-mediated Calpain1/ Erk pathway. LIPUS could be used as an effective physical intervention to alleviate postoperative cognitive dysfunction in the aged population.

## 1. Introduction

With aging accelerating, age-related ailments are becoming a growing concern [1]. The demand for surgery in the elderly is also increasing. Accumulating studies [2,3] have found that anesthesia/surgery (A/S) in the elderly can lead to postoperative cognitive dysfunction, manifested in the early postoperative period as delayed postoperative cognitive recovery (dNCR), which is closely associated with long-term infaust prognosis [4,5], such as long-term cognitive dysfunction and even dementia [6,7]. However, the pathogenesis of postoperative dNCR has yet to be determined [4,8]. It has been confirmed that synaptic dysfunction has a close relationship with cognitive impairment caused by neurodegenerative diseases [9,10,11], but few have been investigated in the elderly suffering postoperative dNCR. Calpain plays a crucial role in maintaining the normal function of the synapse [12]. Previous studies have demonstrated that the neurotrophin brain-derived neurotrophic factor (BDNF) could stimulate hippocampal neurons to activate Calpain1 to proteolyze pleckstrin homology domain and leucine-rich repeat protein phosphatase (PHLPP), which can inhibit the extracellular regulated protein kinases (Erk) pathway, further promote the induction and consolation of long-term potentiation, exerting a neuroprotective effect [13,14,15]. Our previous study found that reversing the decrease in glutamatergic synaptogenesis in aged mice after anesthesia/surgery could be a potential therapeutic target for postoperative dNCR [16]. Beyond that, the prevention and treatment of postoperative dNCR have been extensively explored [17], but effective non-invasive physical interventions are still absent.

Low-intensity pulsed ultrasound (LIPUS), as a non-invasive physical intervention, has been widely used in the past decades to facilitate fracture repair and soft tissue healing [18,19]. Recent studies showed that LIPUS could penetrate the skull and act on a specific region of the brain for precise neuromodulation [20,21,22]. Compared with pharmacological intervention, the LIPUS has many merits, especially avoiding the side effects of systemic administration, which could cause potential damage to the liver or kidney [20]. Shreds of evidence have shown that LIPUS acting on the brain could attenuate neuroinflammation and modulate synaptic plasticity, exerting neuroprotective effects in rodents [23,24], but little has been investigated in dNCR.

It has been proved that LIPUS can act on mechanosensitive ion channels [25]. Piezo is a mechanically sensitive ion channel that contains two isoforms, Piezo1 and Piezo2, of which Piezo1 is widely expressed in the central nervous system [26,27,28]. Recent studies have found that ultrasound can act on Piezo1, and the activation of Piezo1 can lead to the influx of cations such as Ca^2+^ and further regulate downstream signaling pathways [29,30]. Moreover, excessive expression or activation of Piezo1 is closely associated with detrimental statuses, such as aging [31] and infection [32]. Further studies have demonstrated that inhibition of Piezo1/Ca^2+^/calpain signaling could attenuate neuronal apoptosis [33] and improve cognitive impairment [34].

Therefore, we hypothesized that LIPUS application on the hippocampus could promote synaptogenesis and alleviate postoperative dNCR by modulating the Piezo1/Ca^2+^/Calpain1/Erk pathway in aged mice. In this study, a postoperative dNCR model was established to determine whether LIPUS could alleviate hippocampus-dependent memory defect after anesthesia/surgery in aged mice and explore the possible mechanisms.

## 2. Materials and Methods

### 2.1. Animal Care

Aged male C57BL/6 mice (SPF class, 13–14 months) were purchased from Changsha Tianqin Biotechnology Co., Ltd. (Changsha, China) and raised (to 16–18 months old) in the Department of Laboratory Animal Research Center, Peking University, under standard laboratory conditions. All mice were acclimated for 7 days in a controlled environment of 23–25 °C, 50–70% humidity, and a natural light-dark cycle with food and water ad libitum. All animal experiments were performed in accordance with the Guiding Principles for the Care and Use of Animals in Research, the ARRIVE 2.0 guidelines [35], and the protocol was approved by the animal ethics committee of Peking University Health Science Center (No. LA2021568).

### 2.2. Anesthesia/Surgery

**Figure 1:**
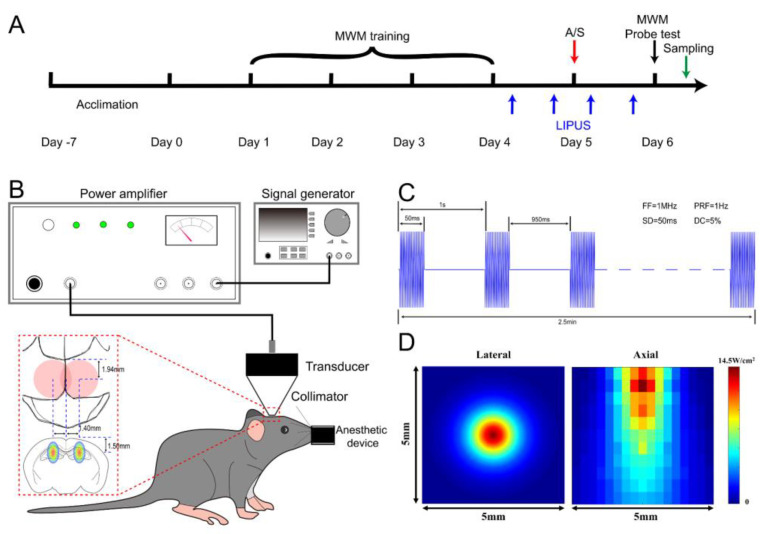
Overview of the LIPUS application. (**A**) Experimental design. (**B**) Diagram of the LIPUS setup and dorsal hippocampus positioning. (**C**) Acoustic intensity distribution of the lateral and axial direction was expressed by the spatial-peak pulse-average intensity (ISPPA). (**D**) Illustration of ultrasound stimulation parameters: fundamental frequency (FF) of 1 MHz, pulse repetition frequency (PRF) of 1 Hz, sonication duration (SD) of 50 ms, and duty cycle (DC) of 5%.

Mice were randomized into four groups: (1) control group (CON), mice received no intervention except anesthesia (described as follows); (2) anesthesia/surgery group (A/S), mice received a laparotomy under sevoflurane anesthesia (induced with 5% sevoflurane for 2–3 min, and maintained with 2.5–3% sevoflurane for 30 min in 40% oxygen). The laparotomy was performed in accordance with previous studies [16,36,37]. Briefly, after disinfection, a 1.5 cm midline incision was made 0.5 cm below the xiphoid, and abdominal organs were explored. Then 5 cm of the small intestinal tissue was removed from the abdominal cavity and rubbed with the fingers for 30 s. The intestine tissue was returned to the abdominal cavity, and then the incision was closed with a 4-0 silk suture. A heating pad was used for body temperature maintenance during the operation. A total of 0.5 mL oxybuprocaine cream was applied every 8 hours to relieve pain after surgery; (3) anesthesia/surgery with LIPUS treated group (A/S + US), mice were treated as A/S group with plus ultrasound intervention; (4) LIPUS treated group (US), mice were the same as CON group but receiving ultrasound treatment. The experimental design is shown in Figure 1A.

### 2.3. LIPUS on Hippocampus

As shown in Figure 1B, the LIPUS was generated by a 1 MHz, focused transducer with 50 ms burst lengths at a 5% duty cycle and a repetition frequency of 1 Hz. Mice were anesthetized (induced with 5% sevoflurane for 2 min and maintained with 2–2.5% sevoflurane in 40% oxygen) and fixed in a brain stereotaxic apparatus (RWD Life Science, Shenzhen, China), then the parietal and occipital skin was shaved, and the surface point of the dorsal hippocampus was marked as bregma (±1.40 mm, caudal−1.94 mm). A calibrating cone was used to adjust the transducer to ensure that the focal point could be on the hippocampus (Figure 1C). The acoustic intensity distribution of the lateral and axial direction of the sonic field is shown in Figure 1D. After transcranial attenuation, the spatial-peak temporal-average intensities (I_SPTA_) 1 mm from the cone tip was 177 mW/cm^2^. The duration of sonication was alternated bilaterally for 2.5 min and repeated three times with a total stimulation time of 15 min. Ultrasound stimulation was conducted at four time points around the anesthesia/surgery: 8 h before anesthesia/surgery, 4 h before surgery, 4 h after surgery, and 8 h after surgery.

### 2.4. Behavioral Test

The Morris water maze is a common method for testing hippocampus-dependent spatial navigation and reference memory in rodents [38]; we conducted this experiment according to previous research with minor modifications [37]. A circular tank (110 cm in diameter and 60 cm in height) was filled with 35 cm of tinted-white water using titanium dioxide and maintained at 24 ± 1 °C, dividing into four quadrants. The invisible platform (10 cm in diameter) was submerged 2 cm below the water’s surface. A training session was performed to allow the mouse to locate the hidden platform. The average time to find the platform was recorded as the escape latency for 4 days prior to the anesthesia/surgery, and if the mouse failed to find the platform for more than 90 s, then the mouse was guided to the platform. On one day postoperatively, the platform was removed, and the mouse was made to swim freely for 90 s. The escape latency, number of platform crossings, and swimming speed were recorded. Data were analyzed using VideoMot2 small animal behavioral activity recording analysis software (TSE System, Berlin, Thuringia, Germany).

### 2.5. Real Time-qPCR

Total RNA was extracted from the dorsal hippocampus using a Trizol reagent (DP424, TianGen, Beijing, China) according to the manufacturer’s protocol. The concentration of total RNA in each sample was measured using a Nano-Drop Spectrophotometer (Thermo Fisher Scientific, Waltham, MA, USA). For mRNA detection, 1000 ng of total RNA was reverse transcribed into cDNA using FastKing First Strand cDNA Synthesis Kit (KR116, TianGen, Beijing, China). Real-time quantitative PCR was performed with SYBR Green Talent qPCR PreMix (FP209, TianGen, Beijing, China) using the QuantStudio 5 Real-Time PCR System (Applied Biosystems Inc., Carlsbad, CA, USA). mRNA expression levels were normalized to the endogenous control β-actin and quantified by the 2^−ΔΔCt^ method. The primer pairs are listed in Table 1.

### 2.6. Immunofluorescent Staining

Mice were transcardially perfused with PBS and then 4% paraformaldehyde after deep anesthesia. Brains were removed and fixed in 4% paraformaldehyde at 4 °C for 24 h. Then the brain slices were made from coronal sections at 30 μm using a cryostat microtome (CM3050S, Leica, Weztlar, Germany). After blocking with normal goat serum (including 0.3% Triton X-100 in PBS) for 1 h at room temperature, the slices were incubated with mouse anti-c-Fos (1:100, ab208942, Abcam, Cambridge, UK) and rabbit anti-Piezo1 (1:100, DF12083, Affinity Biosciences, Zhenjiang Shi, China) at 4 °C overnight. Then the slices were incubated with TRITC-labeled goat anti-mouse (1:200, ZF-0313, ZSGB-Bio, Beijing, China) and FITC-labeled goat anti-rabbit (1:200, ZF-0311, ZSGB-Bio, Beijing, China) secondary antibodies for 1 h at room temperature, stained with DAPI for 10 min at room temperature, and mounted in 70% glycerol. Finally, the slices were imaged using a virtual microscopy slide-scanning system (VS 120, Olympus, Tokyo, Japan). Images of sections containing the dorsal hippocampus were cropped and analyzed using Image J software (V1.48, NIH, Bethesda, MD, USA, 2014).

### 2.7. Hematoxylin-Eosin (HE) Staining

The fixed brain tissues were embedded with paraffin wax, cut into 5-μm slices, carefully attached to slides for drying, and subsequently went through the steps of xylene dewaxing, different concentrations of alcohol to water, hematoxylin and eosin staining, anhydrous ethanol dehydration, xylene transparency and then sealed with treacle, observed and photographed under the light microscope.

### 2.8. Western Blotting

The dorsal hippocampus was separated and homogenized in 200 μL of lysis buffer (R0020, Solarbio, Beijing, China) containing phenylmethanesulfonyl fluoride using a grinder. The lysis buffer was centrifuged at 12,000× *g* for 15 min at 4 °C, and the supernatant was collected. Equal amounts of protein from each sample (20 μg) were separated by SDS-PAGE. The resolved proteins were transferred to polyvinylidene fluoride (PVDF) membranes (IPFL00010, Millipore, Billerica, MA, USA). The membranes were blocked for 1 h in 5% BSA at room temperature and exposed overnight at 4 °C with an appropriate dilution of primary antibodies: rabbit anti-Piezo1 (1:500, 15939-1-AP, Proteintech, Wuhan, China), rabbit anti-BDNF (1:1000, ab108319, Abcam, Cambridge, UK), rabbit anti-synaptophysin(1:1000, ab33127, Abcam, Cambridge, UK), mouse anti-postsynaptic density protein 95 (PSD95) (1:1000, sc-32290, Santa Cruz Biotechnology, Inc., Santa Cruz, CA, USA), rabbit anti-Calpain1 (1:1000, 2556S, CST, Boston, MA, USA), rabbit anti-Calpain2 (1:1000, 2539S, CST, Boston, MA, USA), rabbit anti-PHLPP(1:1000, 22789-1-AP, Proteintech, Wuhan, China), mouse anti-Erk1/2 (1:1000, sc-514302, Santa Cruz Biotechnology, Inc., Santa Cruz, CA, USA ), mouse anti-p-Erk1/2 (1:1000, sc-136521, Santa Cruz Biotechnology, Inc., Santa Cruz, CA, USA ), and mouse anti-β-actin (1:1000, sc-69879, Santa Cruz Biotechnology, Inc., Santa Cruz, CA, USA ). The membranes were then incubated with horseradish peroxidase-conjugated secondary antibody (1:10,000, W4011 or W4021, Promega, Madison, WI, USA) for 1 h at room temperature. All proteins were measured using an enhanced chemiluminescence detection kit (TianGen, Beijing, China, PA112-02) and compared to the loading control protein levels (β-actin) and quantified using Image J (V1.48, NIH, Bethesda, MD, USA, 2014).

### 2.9. Statistical Analysis

GraphPad Prism (V9.0, GraphPad Software, La Jolla, CA, USA, 2020) was used to analyze the data. Data were presented as means ± SEM. The normal distribution of data was tested with the Kolmogorov-Smirnov test (*p* > 0.1). One–way ANOVA followed by Bonferroni’s multiple comparisons test was used for analyzing the data among the four groups. A two-way repeated-measures ANOVA followed by a post hoc Bonferroni test were used to analyze the water maze escape latency during the training session. A *p*-value less than 0.05 was considered statistically significant.

## 3. Results

### 3.1. LIPUS Could Safely Induce Neuronal Activation in Dorsal Hippocampus

**Figure 2:**
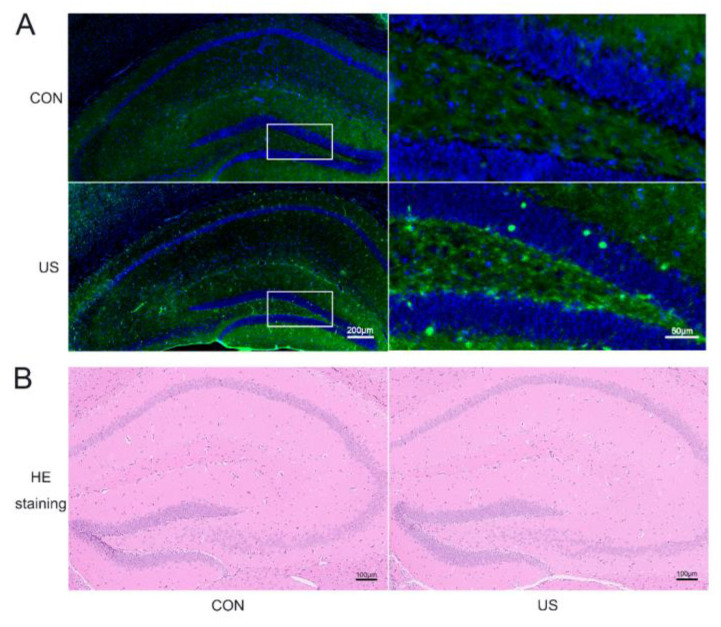
LIPUS can safely and precisely act on the dorsal hippocampus. Immunofluorescence staining of c-Fos (**A**) and HE stain (**B**) of the dorsal hippocampus with or without US treatment. CON: The sham group; US: The US treated group for 15 min.

To investigate whether the LIPUS could affect the neural cells of the dorsal hippocampus, c-Fos, a classical marker of neuronal activation [39], was selected to incubate the brain slices after the LIPUS exposure. As shown in Figure 2A, the c-Fos expression significantly increases, suggesting that LIPUS indeed activated the dorsal hippocampus. Moreover, the HE staining was used to evaluate whether our LIPUS did harm to the mice. As shown in Figure 2B, there were no obvious changes in the exposure group compared with the CON group.

### 3.2. LIPUS Attenuates Hippocampus-Dependent Spatial Reference Learning and Memory Injury after A/S in Aged Mice

**Figure 3:**
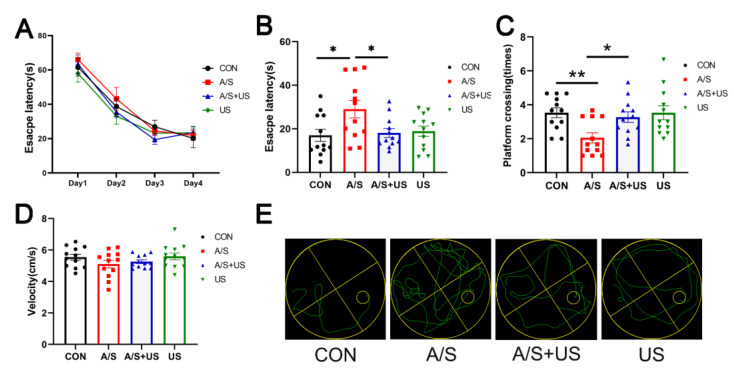
LIPUS improves hippocampus-dependent learning and memory after anesthesia/surgery in aged mice. (**A**) Escape latency in the training stage before anesthesia/surgery. (**B**) Escape latency one day after LIPUS application. (**C**)Platform crossing times 1 day after anesthesia/surgery after LIPUS application. (**D**) Swimming speed of four groups during the process of testing. (**E**) The representative swimming trajectories of the four groups during the Morris water maze test. *n* = 12 per group. Data were presented as Mean ± SEM. * *p* < 0.05, ** *p* < 0.01.

On the first postoperative day, the escape latency of the A/S group was significantly extended compared with the CON group; while the escape latency of the A/S + US group was obviously shortened than that of the A/S group and was approximately equal to the CON group (Figure 3B). Moreover, the mice in the A/S + US group showed more platform crossing times than that of the A/S group, and there was no difference among the CON group, A/S + US, and US groups when the platform was removed (Figure 3C). In addition, as shown in Figure 3A,D, there was no difference in the basic cognitive level and swimming speed among the four groups. In summary, anesthesia/surgery could impair hippocampus-dependent spatial reference learning and memory, while the LIPUS could effectively attenuate those impairments.

### 3.3. LIPUS Reduces the Neuroinflammation of Hippocampus after A/S in Aged Mice

**Figure 4:**
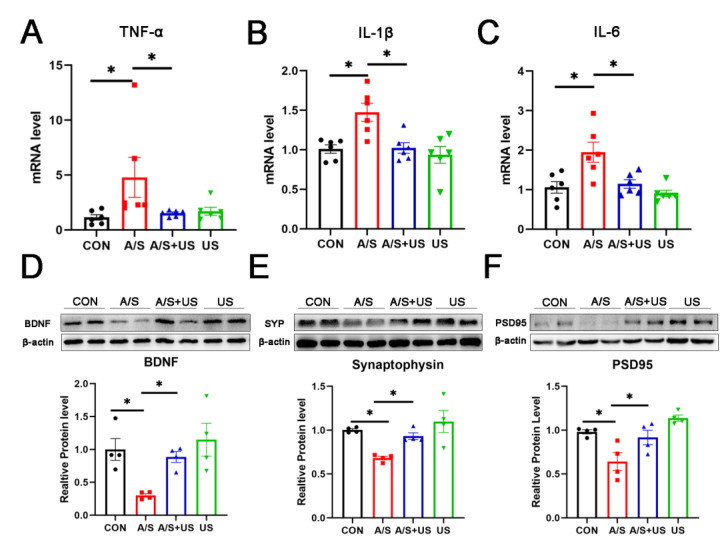
LIPUS attenuates neuroinflammation and synaptic dysfunction of the hippocampus after anesthesia/surgery in aged mice. The mRNA level of inflammatory cytokines of TNF-α (**A**), IL-1β (**B**), and IL-6 (**C**) in the hippocampus among the four groups (*n* = 6 per group). Western blotting analysis of the synapse-related proteins BDNF (**D**), Synaptophysin (**E**), and PSD95 (**F**) in the hippocampus among the four groups (*n* = 4 per group). Data were presented as Mean ± SEM. One-way ANOVA followed by Bonferroni’s multiple comparisons test. * *p* < 0.05.

Consistent with our previous studies (21, 46), after anesthesia/surgery, the mRNA level of hippocampal proinflammatory factors (TNF-α, IL-1β, and IL-6) increased in aged mice compared with the CON group (Figure 4A–C). LIPUS intervention could effectively reduce the neuroinflammation associated with anesthesia/surgery in the hippocampus. It is worth noting that LIPUS application alone did not increase neuroinflammatory responses in the hippocampus compared to the CON group, which supports the view that our parameter of LIPUS was safe (Figure 4A–C).

### 3.4. LIPUS Reverses the Reduced Synapse-Related Proteins of the Hippocampus after A/S in Aged Mice

BDNF plays an important role in maintaining the normal function of the synapse [40]. Compared with the CON group, BDNF was decreased in the A/S group, and LIPUS alleviated this decline in the AS + US group (Figure 4D). As landmark proteins located in the pre- and post-synapse, synaptophysin and PSD95 are widely involved in synaptic information transmission [11,41]. Similarly, the A/S group showed significant decreases in synaptophysin and PSD95, while the LIPUS obviously rescued this decreased protein expression in the AS + US group (Figure 4E,F). No difference in BDNF, synaptophysin, or PSD95 was detected between the CON group and the US group (Figure 4D–F).

### 3.5. LIPUS Suppresses the Overexpression of Piezo1 after A/S in Aged Mice

**Figure 5:**
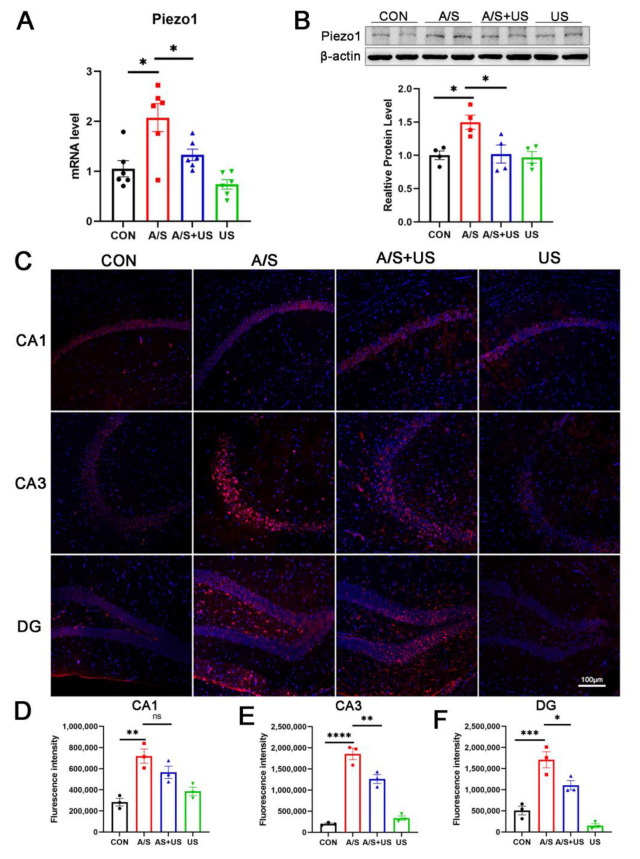
LIPUS inhibits Piezo1 expression after anesthesia/surgery in aged mice. (**A**) The mRNA expression of Piezo1 was qualified by RT-qPCR among the four groups (*n* = 6 per group). (**B**) Western blotting detection of Piezo1 among the four groups (*n* = 4 per group). (**C**) Immunofluorescence detection of Piezo1 expression in CA1 (**C**,**D**), CA3 (**C**,**E**), and DG (**C**,**F**) regions among the four groups (*n* = 3 per group). Data were presented as Mean ± SEM. One-way ANOVA followed by Bonferroni’s multiple comparisons tests. * *p* < 0.05, ** *p* < 0.01, *** *p* < 0.001, **** *p* < 0.0001.

It has been confirmed that LIPUS can interact with piezo1 receptors, but the effect of different ultrasound stimulation intensities on Piezo1 remains unclear. In this study, we found that the mRNA and protein level of Piezo1 expression was elevated after anesthesia/surgery in aged mice compared to the CON group (Figure 5A,B). LIPUS reduced the overexpression of Piezo1 associated with the A/S in the AS + US group. The results of immunofluorescence further showed a significant increase in Piezo1 after anesthesia/surgery (Figure 5C–F); a significant downregulation of Piezo1 in the CA3 (Figure 5C,E) and DG (Figure 5C,F) regions, but not in the CA1(Figure 5C,D) region, was observed after LIPUS application.

### 3.6. LIPUS May Improve Synaptic Function through Modulating Piezo1/Calpain/Erk Pathway

**Figure 6:**
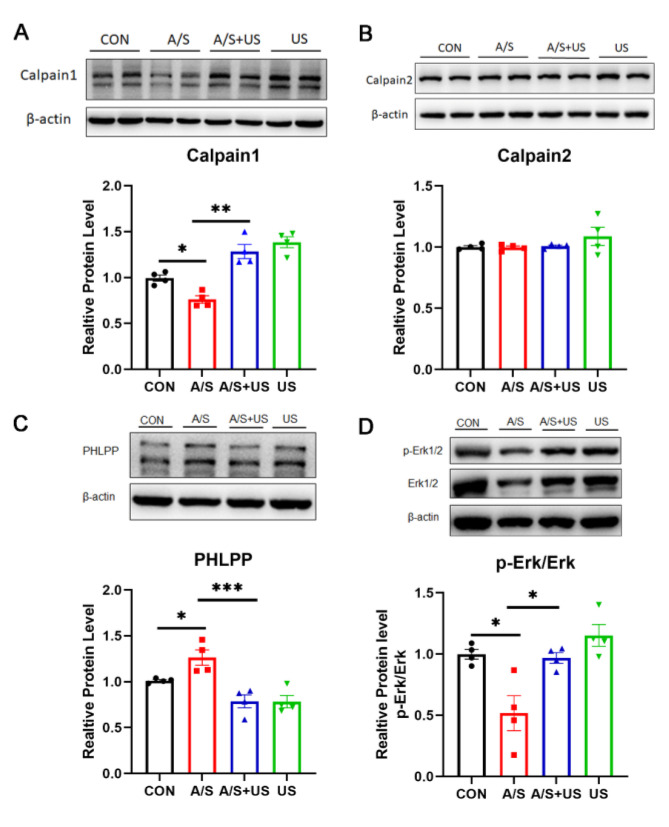
LIPUS may modulate the calpain1/Erk pathway to salvage synaptogenesis after anesthesia/surgery in aged mice. Western blotting analysis of Calpain1 (**A**) and Calpian2 (**B**) expression among the four groups. (**C**) PHLPP, as the substrate of calpain1, was used to represent the activity of calpain1. (**D**) Erk phosphorylation level was expressed as the ratio of p-Erk to total Erk and normalized to the internal control β-actin. *n* = 4 per group. Data were presented as Mean ± SEM. One-way ANOVA followed by Bonferroni’s multiple comparisons tests. * *p* < 0.05, **p < 0.01, *** *p* < 0.001.

The calpain protease system and its downstream Erk pathway play an important role in the regulation of synaptic function [12,42]. In our study, after the A/S, hippocampal Calpain1 expression, but not Calpain2, was decreased, and the content of PHLPP (an important calpain1 substrate) was higher than that of the CON group (Figure 6A–C), suggesting that A/S could reduce the activity of Calpian1. When receiving the intervention of LIPUS, the changed tendency of hippocampal Calpain1 and PHLPP in the A/S + US group was reversed compared with the A/S group (Figure 6A,C), suggesting that LIPUS was able to enhance the activity of Calpian1. Erk has been proven to be a classical downstream pathway of Calpain1 [12]. The ratio of its phosphorylation form (p-Erk/Erk) in the A/S group was decreased compared with the CON group, while the LIPUS reversed this trend in the A/S + US group (Figure 6D), which means a possible mechanism of LIPUS’ improving dNCR is activating the Erk pathway.

## 4. Discussion

As an emerging and non-invasive tool, it has been demonstrated that LIPUS has many advantages in neuromodulation [22]. With the help of low-frequency and focused acoustic waves, the LIPUS could penetrate the skull with high spatial resolution, which enables a precise application to target areas inside the skull for neuromodulation [21,43]. Our results showed that LIPUS could activate neural cells in the hippocampus, and no obvious changes were detected between the CON group and the LIPUS group, suggesting that LIPUS application on the dorsal hippocampus is effective and safe under current parameters.

Postoperative dNCR, manifested as cognitive and behavioral changes within 30 days after anesthesia/surgery, plays a pivotal role in the occurrence and development of PND. However, there is currently no normative term for postoperative cognitive research in animals. Several scholars proposed that “abnormal changes in neurocognitive behavior (Morris water maze or fear condition test) lasting 1–2 weeks after anesthesia/surgery in rodents should be attributed to postoperative dNCR” [44]. In this study, the Morris water maze test showed the escape latency was significantly increased, while LIPUS intervention could shorten it significantly, indicating that LIPUS intervention could improve postoperative dNCR in aged mice. Meanwhile, LIPUS intervention reduced elevated levels of inflammatory factors in the hippocampus after anesthesia/surgery in aged mice. This similar anti-inflammatory effect is consistent with previous studies [23,45].

Many investigations have been conducted to explore the pathogenesis of postoperative dNCR, including neuroinflammation [46], blood-brain barrier disruption [47], imbalance of neurotransmitters and receptors in the brain [48], impaired energy metabolism [49], aggregation of abnormal proteins [50], and neuronal synaptic dysfunction [16,51]. In previous studies, LIPUS has been used for the prevention and treatment of several neurodegenerative diseases and cerebrovascular diseases [52,53,54]. However, the exact mechanism remains poorly elucidated. In this study, LIPUS was found to improve postoperative dNCR in aged mice, probably through modulating the Piezo1 receptor and salvaging synaptic function.

**Figure 7:**
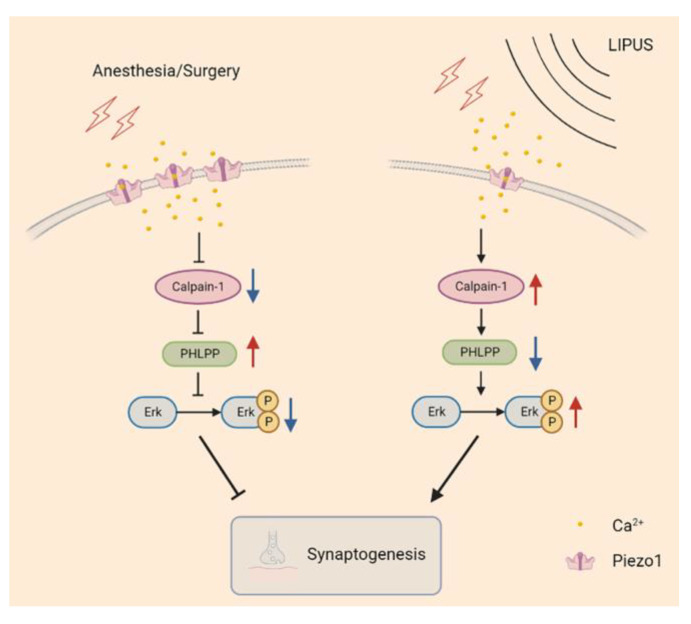
The possible signaling pathway of LIPUS mediated neuroprotection after anesthesia/surgery in aged mice. Piezo1 expression was increased in aged mice undergoing anesthetic surgery, and an excessive influx of Ca^2+^ inhibited calpain1 activity, manifested as reduced PHLPP degradation, further alleviated Erk phosphorylation, and led to synapse dysfunction (left column). In contrast, LIPUS application inhibited Piezo1 expression and enhanced calpain1 activity, promoting PHLPP degradation, which released the inhibition of Erk phosphorylation and rescued synaptic function (right column).

Piezo1, an ion channel capable of sensing mechanical stress, has been shown to be activated by ultrasonic waves, leading to conformation changes and producing certain biological effects [29,30]. Wang et al. found that the content of Piezo1 increased both in vivo (in rat cerebral cortex after the ischemia/reperfusion in the intraluminal middle cerebral artery occlusion model) and in vitro (in an oxygen-glucose deprivation/reoxygenation injury cell model) accompanied by decreasing in-cell viability and increasing in-cell apoptosis; on the contrary, these biological effects were reversed when the Piezo1 was inhibited [33]. In our study, the Piezo1 receptors were increased in the hippocampus after A/S in aged mice; and the LIPUS effectively suppressed this trend and attenuated hippocampus-dependent spatial reference learning and memory injury, which suggested the Piezo1 receptors might play an important role in the process of LIPUS’ beneficial to postoperative dNCR. It is reported that piezo1 expression was upregulated after cells received noxious stimuli and then promoted excessive intracellular Ca^2+^ aggregation [31,33]. We speculated that the upregulation of the Piezo1 receptor led to a large intracellular Ca^2+^ accumulation, leading to the inhibition of Calpain1 activity. Calpain1 is vital in the maintenance of synaptic function in the CNS, and Calpain1 activation degrades PHLPP [15]. The PHLPP could inhibit the Erk pathway, thereby improving synaptic functions [42,55]. In this study, after the LIPUS application, the trend of Calpain1 and PHLPP induced by the A/S was reversed, which may relieve the inhibition of the downstream Erk pathway and further mitigate synaptic dysfunction (Figure 7).

BDNF, synaptophysin, and PSD95 contribute to the maintenance of normal synaptic morphology and function [40,41,56]. Our study found that BDNF expression was downregulated in the hippocampus of aged mice after anesthesia/surgery, and ultrasound intervention could promote BDNF expression, which is consistent with previous literature [23,57]. Our study found that hippocampal synaptophysin and PSD95 expression were reduced after anesthesia/surgery in aged mice, while LIPUS increased synaptophysin and PSD95. Taken together, LIPUS may alleviate the reduction of BNDF, synaptophysin, and PSD95 induced by the anesthesia/surgery in aged mice, but whether LIPUS affects the structure and function of synapses needs further study.

Limitations still exist in this study. Firstly, we found the amount of Piezo1 receptors might be associated with the LIPUS’ beneficial effect on dNCR. However, whether the function of Piezo1 receptors is altered under the effect of ultrasound needs to be further explored. Secondly, only the hippocampus was investigated as the effect of anesthesia/surgery is systemic. However, it has been proven by numerous studies that the hippocampus was the key zone for spatial reference learning and memory, especially for the Morris water maze. Finally, although it has been demonstrated that the Piezo1 could regulate the downstream calpain1/Erk pathway in other models, the concrete relationship still needs to be determined in the dNCR.

## 5. Conclusions

In summary, our study found that LIPUS could improve delayed postoperative cognitive recovery, reduce neuroinflammation in the hippocampus, and salvage hippocampal synaptogenesis in aged mice, which may be related to modulating the Piezo1/calpain1/Erk pathway, but the exact mechanism needs further study. Our study also provides a new insight into dNCR prevention and treatment with the neuromodulation of LIPUS.

## Figures and Tables

**Table 1:** Primers Sequences for RT-qPCR.

Gene ID	Protein Name	Forward	Reverse
*Tnf*	TNF-α	ATGTCTCAGCCTCTTCTCATTC	GCTTGTCACTCGAATTTTGAGA
*Il1b*	IL-1β	CACTACAGGCTCCGAGATGAACAAC	TGTCGTTGCTTGGTTCTCCTTGTAC
*Il6*	IL-6	CTCCCAACAGACCTGTCTATAC	CCATTGCACAACTCTTTTCTCA
*Piezo1*	Piezo1	GAATGTGATTGGGCAGCGTATGAAC	GAACAGCGTGAGGAACAGACAGTAG
*Actb*	β-actin	TATGCTCTCCCTCACGCCATCC	GTCACGCACGATTTCCCTCTCAG

## Data Availability

The datasets used and/or analyzed during the current study are available from the corresponding authors upon reasonable request.
